# Genome Sequence of the Polyphosphate-Accumulating Organism *Arthrobacter* sp. Strain PAO19 Isolated from Maize Rhizosphere Soil

**DOI:** 10.1128/genomeA.00566-13

**Published:** 2013-08-01

**Authors:** Xiaolong Li, Hongli Yuan, Jinshui Yang, Baozhen Li

**Affiliations:** State Key Laboratory of Agrobiotechnology, Ministry of Agriculture, Key Laboratory of Soil Microbiology, College of Biological Sciences, China Agricultural University, Beijing, China

## Abstract

*Arthrobacter* sp. strain PAO19 is a polyphosphate-accumulating organism isolated from maize rhizosphere soil. Here we report its genome sequence, which may shed light on its role in phosphate removal from water bodies. To our knowledge, this is the first genome announcement of a polyphosphate-accumulating strain of the genus *Arthrobacter*.

## GENOME ANNOUNCEMENT

Eutrophication, mainly caused by overmuch nitrogen (N) and phosphorus (P) nutrients, is one of the most serious water pollution problems (1). N limitation, however, strengthened the competitive advantage of nitrogen-fixing cyanobacteria and they replenished the N pool of water bodies (2). So P control is crucial to mitigating eutrophication (3, 4). Polyphosphate-accumulating organisms (PAOs) uptake surplus orthophosphate (Pi) and accumulate it as inorganic polyphosphate (polyP) in cells (5). PAOs play pivotal roles in biological phosphate removal (BPR) from wastewater and rehabilitation of eutrophic water bodies (6). One efficient bacterium, isolated in our lab from maize rhizosphere soil and designated strain PAO19, removed 92% of P from synthetic wastewater with 10 mg/liter of Pi (our unpublished data). The 16S rRNA gene of strain PAO19 (GenBank Accession no. JN676107) displays high identity to that of *Arthrobater*, with maximum identity to that of *A. nicotianae* strain DSM 20123. *Arthrobacter* spp. strains are among the most frequently isolated, indigenous, aerobic bacterial genera found in soils. Members of the genus are metabolically and ecologically diverse and have the ability to survive in environmentally harsh conditions for extended periods of time (7, 8). To date, little has been reported on BPR and polyP accumulation by *Arthrobacter* spp. strains (9, 10). In addition, few genomes of PAOs have been sequenced and announced so far (11, 12). In this study, the genome of *Arthrobacter* sp. strain PAO19 was determined to provide novel insight into the molecular mechanism of microbial P metabolism and polyP accumulation. This work has the potential to help promote effective control of eutrophication of water bodies in future practice.

The genome sequencing of *Arthrobacter* sp. strain PAO19 was performed using an Illumina Hiseq2000 with 101-bp paired-end reads. The reads were trimmed and assembled *de novo* using SOAPdenovo2 (13). The prediction of open reading frames (ORFs), tRNAs, and rRNAs was performed by using Glimmer 3.02, tRNAscan-SE 1.3, and RNAmmer 1.2 (14–16), respectively. Subsequent genome annotation was done using Blast2GO 2.5.0 (17). The *de novo* assembly yielded 499-fold coverage of a 3,637,746-bp draft genome contained in 47 contigs with an average GC content of 59.52%. The contig *N*_50_ was approximately 246 kb, and the largest contig assembled was approximately 448 kb. A total of 3,451 ORFs, 41 tRNAs, and 3 rRNAs were predicted from the draft genome.

In functional annotation of the genome, we identified as being involved in the metabolisms of phosphorus and inorganic polyphosphate the genes for polyphosphate kinase domain-containing protein at contig 1 and contig 8, polyphosphate kinase and exopolyphosphatase at contig 1, polyphosphate-glucose phosphotransferase at contig 9, low-affinity inorganic phosphate transporter at contig 11, phosphate ABC transporter substrate-binding protein PstS at contig 7 and contig 23, phosphate ABC transporter inner-membrane subunits PstC and PstA and phosphate ABC transporter ATP-binding subunit PstB at contig 19, phosphate transport system regulatory protein PhoU at contig 24, and alkaline phosphatase at contig 6.

### Nucleotide sequence accession numbers. 

This whole-genome shotgun project has been deposited in DDBJ/EMBL/GenBank under the accession no. ATKN00000000. The version described in this paper is the first version, ATKN01000000.
